# Experimental blunt chest trauma-induced myocardial inflammation and alteration of gap-junction protein connexin 43

**DOI:** 10.1371/journal.pone.0187270

**Published:** 2017-11-09

**Authors:** Miriam Kalbitz, Elisa Maria Amann, Belinda Bosch, Annette Palmer, Anke Schultze, Jochen Pressmar, Birte Weber, Martin Wepler, Florian Gebhard, Hubert Schrezenmeier, Rolf Brenner, Markus Huber-Lang

**Affiliations:** 1 Department of Traumatology, Hand-, Plastic-, and Reconstructive Surgery, Center of Surgery, University of Ulm, Ulm, Germany; 2 Institute of Clinical Transfusion Medicine and Immunogenetics Ulm, German Red Cross Blood Transfusion Service Baden-Württemberg–Hessen and University Hospital Ulm, University of Ulm, Ulm, Germany; 3 Institute of Clinical and Experimental Trauma-Immunology, University of Ulm, Ulm, Germany; 4 Institute of Anaesthesiological Pathophysiology and Process Engineering, University of Ulm, Ulm, Germany; 5 Division of Biochemistry of Joint and Connective Tissue Diseases, Department of Orthopedics, University of Ulm, Ulm, Germany; Indiana University School of Medicine, UNITED STATES

## Abstract

**Objective:**

Severe blunt chest trauma in humans is associated with high mortality rates. Whereas lung tissue damage and lung inflammation after blunt chest trauma have extensively been investigated, the traumatic and posttraumatic effects on the heart remain poorly understood. Therefore, the purpose of this study was to define cardiac injury patterns in an experimental blunt chest trauma model in rats.

**Methods:**

Experimental blunt chest trauma was induced by a blast wave in rats, with subsequent analysis of its effects on the heart. The animals were subjected either to a sham or trauma procedure. Systemic markers for cardiac injury were determined after 24 h and 5 days. Postmortem analysis of heart tissue addressed structural injury and inflammation 24 h and 5 days after trauma.

**Results:**

Plasma levels of extracellular histones were elevated 24 h and 5 days after blunt chest trauma compared to sham-treated animals. In the heart, up-regulation of interleukin-1β 24 h after trauma and increased myeloperoxidase activity 24 h and 5 days after trauma were accompanied by reduced complement C5a receptor-1 expression 24 h after trauma. Histological analysis revealed extravasation of erythrocytes and immunohistochemical analysis alteration of the pattern of the gap-junction protein connexin 43. Furthermore, a slight reduction of α-actinin and desmin expression in cardiac tissue was found after trauma together with a minor increase in sarcoplasmatic/endoplasmatic reticlulum calcium-ATPase (SERCA) expression.

**Conclusions:**

The clinically highly relevant rat model of blast wave-induced blunt chest trauma is associated with cardiac inflammation and structural alterations in cardiac tissue.

## Introduction

In the United States, approximately 30,000 patients with blunt cardiac trauma were recorded annually [1]. Heart injury represents an independent predictor for a poor outcome [2], prolonged ventilation interval [3] and longer hospital stay [4], and is associated with dysrhythmias (including ventricular fibrillation) and sudden cardiac arrest [5, 6]. Accordingly, myocardial contusion strongly correlates with the incidence of perioperative hemodynamic instability [7]. In the experimental setting of blast injury, bradycardia [8, 9] and decreased mean arterial pressure [10] [8, 11] have been reported. Some studies have postulated a reflexive nature of the blast wave-induced bradycardia, which could be prevented by vagotomy [11] or traumatic stimulation of arterial baroreceptors [12]. In addition, effects of blunt cardiac injury appear to enhance the firmness of cardiac tissue, as demonstrated in a pig model of blunt chest trauma [13].

In general, following tissue damage, endogenous alarmins are released, which may interact with cellular receptors to induce a proinflammatory immune response [14, 15]. In this context, circulating nucleosomes have been reported to correlate with the injury severity score in humans. Furthermore, they may dissociate to individual histones [16], which in turn interact with a variety of cells, including cardiomyocytes [17–19]. During systemic inflammation, the occurrence of histones has been linked to organ failure and mortality [16, 19–21]. Following major trauma in humans, a series of plasma biomarkers, including interleukin (IL)-6 [22] and the anaphylatoxins C3a and C5a [23], are elevated. Interaction of C5a with the C5a receptor 1 (C5aR1) can induce dramatic contractile dysfunction in cardiomyocytes in vitro [24] and in vivo during sepsis.

However, the mechanistic basis of depressed cardiac function after trauma remains elusive. Therefore, in the present study, a blast wave-induced blunt chest trauma model was applied to investigate cardiac injury.

## Materials and methods

### Animals

All experimental and animal procedures followed the guide of care and use of laboratory animals published by the US National Institutes of Health. The study was approved by the regional animal care and use committee (Regierungspräsidium Tübingen) and performed according to guidelines (Permit No. 1182). Adult, male Wistar rats (7–8 weeks, 310 ± 50 g) were obtained from Charles River (Sulzfeld, Germany). All animals had free access to water and food before and after chest trauma.

### Animal preparation

Rats were anesthetized using a 2.5% sevoflurane (Abott, Wiesbaden, Germany) and 97.5% oxygen mixture under continuous flow of 1 l/min. Buprenorphine (Essex, Pharma, Munich, Germany) was administered subcutaneously (0.03 mg/kg body weight) immediately after trauma or sham procedure and 8 h thereafter.

### Blunt chest trauma

Bilateral lung contusion was induced by a single blast injury in anesthetized rats as described previously [10, 25, 26]. In brief, opening of a high-speed valve (Hee-D-24, Festo, Esslingen, Germany) delivered compressed air into the upper section of a cylinder. The upper compartment was separated from the lower compartment by a Mylar® polyester film (Du Pont de Nemur, Bad Homburg, Germany). The polyester membrane ruptured at a standardized pressure, thus releasing a defined blast wave in the lower section of the cylinder, centered on the ventral thorax of the animal. The level of the blast impact to induce pulmonary contusion was chosen based on histologic, cardiopulmonary and immunologic changes in earlier studies, but without lethal outcome. Sham procedure included anesthesia and analgesia without blunt chest trauma. After trauma or sham procedure the animals were monitored every 8 h regarding bodyweight, breathing, vigilance and mobility as well as general state of health. Buprenorphine (Essex) was administered subcutaneously (0.03 mg/kg body weight) immediately after trauma or sham procedure and 8 h thereafter. At the end of the observation period the animals were anesthetized using a 2.5% sevoflurane (Abott, Wiesbaden, Germany) and 97.5% oxygen mixture under continuous flow of 1 l/min and exsanguinated.

### Sample collection

Full-blood samples were collected 24 h and 5 d after trauma (S-Monovette EDTA, SARSTEDT AG, Nümbrecht, Germany) and kept at 4°C for 40 min,. After centrifugation (800x*g* for 5 min at 4°C followed by 13,000x*g* for 2 min at 4°C), plasma was removed and stored at −80°C until analysis. Heart tissue of the left ventricles was obtained either at 24 h or 5 d after blunt chest trauma and fixed using 3.7% formalin followed by embedding in paraffin until analysis.

### Histone enzyme-linked immunosorbent assay (ELISA)

Histones in rat plasma were determined using a cell-death detection ELISA kit, which detects all histones (Hoffmann-La Roche, Indianapolis, IN). A histone mixture (containing H2, H2A, H2B, H3, H4) was used to establish a standard curve, as described previously [21].

### Western blotting

Left ventricular tissue was obtained 24 h or 5 d after multiple trauma or sham procedure and homogenized and lysed using RIPA Lysis Buffer (EMD Millipore, Darmstadt, Germany) containing complete Mini-protease inhibitor and PhosSTOP protease inhibitor cocktail (Roche). Protein concentrations were determined in homogenates using the Pierce^®^ BCA Protein Assay Kit (Thermo Fischer Scientific, Waltham, MA, USA). Samples were loaded under reducing conditions onto a Mini-Protean®TGX™ Precast Gel (Bio-Rad Laboratories, Munich, Germany). After electrophoresis, the proteins were transferred by a Trans-Blot Turbo Transfer System using Mini Transfer Packs (Bio-Rad). The blots were blocked with 5% milk (C5aR1) or 5% bovine serum albumin (connexin 43 (Cx43)) in tris-buffered saline for 1 h at room temperature (RT) and incubated with antibodies (see below) overnight at 4°C. For analysis of the rat heart homogenates, rabbit anti-C5aR1 (Proteintech, Manchester, UK) and for Cx43 rabbit anti-connexin (Cell Signaling Technology, Danvers, MA) were used. HRP-conjugated anti-rabbit immunoglobulin G (IgG) (Cell Signaling) was used as a secondary antibody. Chemiluminescent HRP Hy Glo™ (Denville Scientific Inc, South Plainfield, NJ) was used. The blots were analyzed by ChemiDoc (BioRad Laboratories GmbH, Munich, Germany) and Image Lab Software (Version 5.2, BioRad).

### Detection of mRNA for IL-1β, DES, ACTN2 and ATP2A2 in rat-heart homogenates by real-time quantitative PCR (qRT-PCR) analysis

Rat left ventricles were obtained 24 h or 5 d after thorax trauma or sham procedure. Total RNA was isolated from rat heart homogenates by the TRIZOL^®^ method (Thermo Fischer Scientific) according to manufacturer’s instructions. cDNA was generated and amplified (SYBR^®^) using reagents from Life Technologies. Amplification was performed using Stratagene Mx 300P (Thermo Fischer Scientific, Waltham, MA, USA). Calculation of the relative quantitative results was performed with the 2^−ΔΔCt^ algorithm. The following primers were used:

Il-1β 5'GCCCTTGACTTGGGCTGTCC3' (forward),

Il-1β 5'GAAGCTCCACGGGCAAGACA3' (reverse);

desmin (DES) 5'TGTTCCCACGAGCAGGCTTC3' (forward),

DES 5'CCTGGTACACGCGGGATGTC3' (reverse);

actinin alpha 2 (ACTN2) 5'AGCGGCAGTCCATCCTAGCC3' (forward),

ACTN2 5'GCAGCTCGTCCATGGTGACA3' (reverse);

ATPase, Ca^2+^ transporting, cardiac muscle, slow twitch 2 (ATP2A2) 5'CTGACCCTGTCCCTGACCCA3' (forward),

ATP2A2 5'CGCCACCACCACTCCCATAG3' (reverse);

C5aR1 5‘-TTGTGCGTTTCCCTGGCCTA-3‘ (forward),

C5aR1 5‘-GCTGTCCCTGCCCAAGGAGT-3‘ (reverse).

Housekeeping gene hypoxanthine phosphoribosyltransferase 1 (HPRT 1) 5'AGGACCTCTCGAAGTGTTGG3' (forward),

HPRT 1 5'TTTCCACTTTCGCTGATGAC3' (reverse).

### Histology by hematoxylin and eosin and immunohistochemistry

To determine extravasal bleeding in cardiac muscle, 4-μm paraffin sections were stained with Gill’s hematoxylin and eosin (H.E, Morphisto Evaluationsforschung & Anwendung GmbH, Frankfurt am Main, Germany). The formalin-fixed left ventricles were immunohistochemically analyzed. A heat-induced antigen retrieval was performed by boiling the sections twice in sodium citric buffer (pH 6). C5aR1 was detected using a polyclonal rabbit anti-C5aR1 antibody (MyBioSource, San Diego, CA, USA). Caspase 3 staining was performed by rabbit anti-cleaved caspase 3 (Cell Signaling, Danvers, MA, USA). After incubation with the primary antibody, detection by DAKO Real Detection System alkaline phosphatase red (Dako, Glostrup, Denmark) was performed. The sections were counterstained with Mayer’s hematoxylin (Sigma, Darmstadt, Germany). Control staining was performed using a nonspecific rabbit IgG (Dako). For Cx43 localization in left ventricular cardiomyocytes, rabbit anti-Cx43 (Cell Signaling Technology) was used.

Apoptosis staining was conducted using the CF488A TUNEL apoptosis detection kit (Biotium, Fremont, USA) according to the manufacturer’s protocol. Following staining, cells were washed twice with PBS and covered with ProLong® Gold Antifade Mountant with 4´6-Diamidin-2phenylindol (DAPI) (Thermo Scientific). For imaging, an Axio Imager M.1 Microscope (Carl Zeiss AG, Oberkochen, Germany) was used. Pixel density was determined using Image J [27].

### Heart myeloperoxidase (MPO)

Heart MPO activity was quantified as described previously [28]. In brief, left ventricular tissue was homogenized (Ultraturrax T25, Jahnke und Kunkel, Staufen, Germany) in 1.5 ml buffer containing 10.35 g KH_2_PO_4_ (Merckmillipore, Darmstadt, Germany) in 950 mldistilled water adjusted to pH 5.4 using 0.91 g K_2_HPO_4_ (Merckmillipore) in 50 ml distilled water and 0.5% hexadecyltrimethylammonium bromide (Sigma). Tissue homogenates were incubated at 60°C for 2 h followed by centrifugation at 3,950x *g* at RT for 15 min. A total of 25 μl tissue or standard solution (Novabiochem, Schwalbach, Germany) was mixed with 25 μl tetramethylbenzidine (Sigma) and 200 μl 0.002% H_2_O_2_ (Fluka, Deisenhofen, Germany) and incubated at 37°C for 5 min. Extinction values were read at 450 nm and MPO activity was calculated on the basis of standard curves. Thereafter, perchloric-acid precipitation was performed and the protein concentration was determined using the Pierce® BCA Protein Assay Kit (Thermo Scientific) as recommended by the manufacturer.

### Statistical analysis

All values were expressed as means ± standard error of the mean. Data were analyzed by one-way analysis of variance followed by Dunnett’s multiple comparison test. A value of p≤0.05 was considered statistically significant. GraphPad Prism 7.0 software was used for statistical analysis (GraphPad Software, Incorporated, San Diego, Ca, USA).

## Results

### Appearance of extracellular histones in plasma and extravasation of erythrocytes in cardiac tissue after blunt chest trauma (Fig 1)

Because there is evidence that extracellular histones are elevated in human plasma after trauma [16] and because cardiac dysfunction has been linked to the appearance of extracellular histones during sepsis [29], plasma levels of extracellular histones were determined. The plasma extracellular-histone concentration was elevated 24 h and 5 d after trauma compared to sham-treated animals.

**Fig 1:**
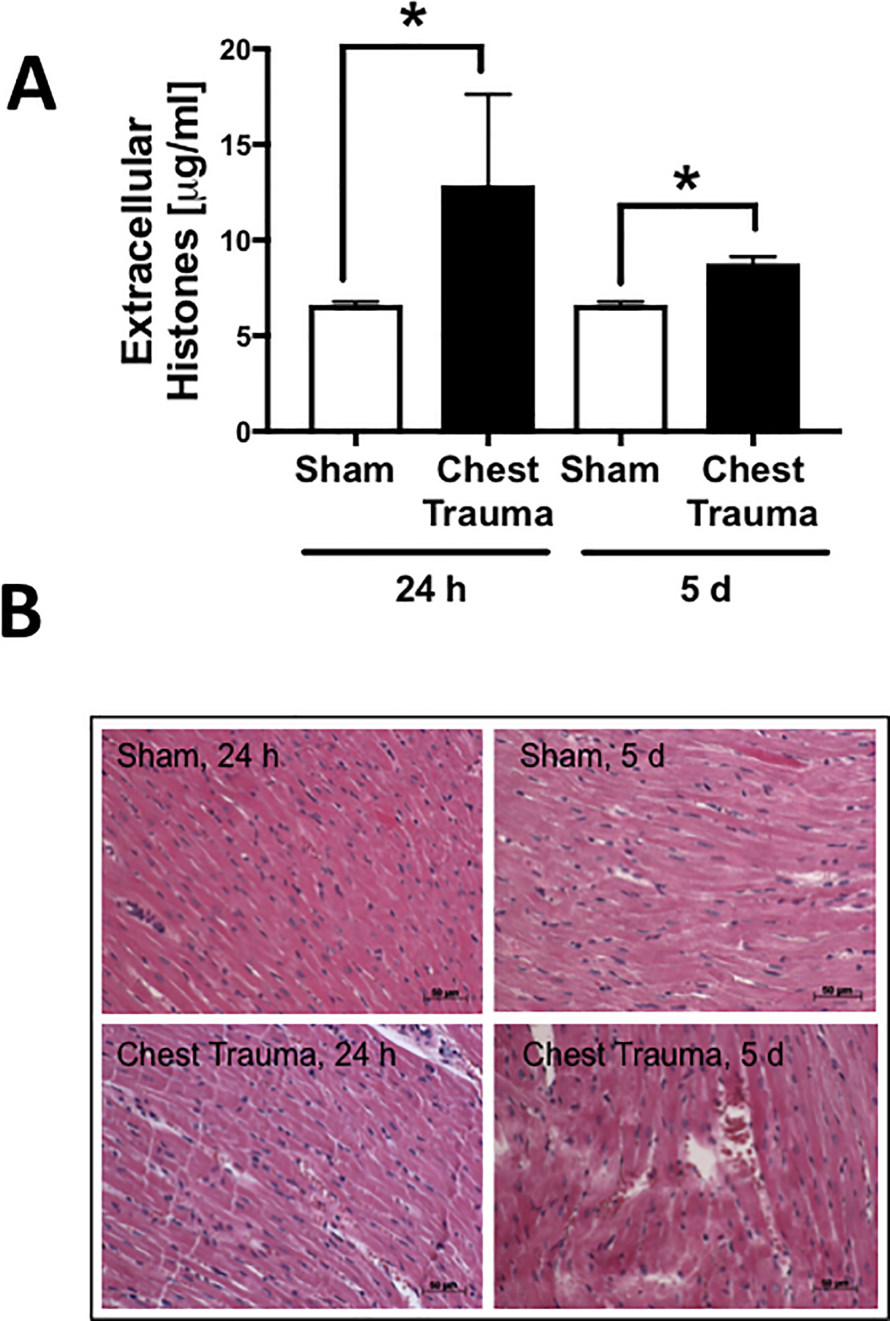
Systemic and local effects of blunt chest trauma. Apparence of extracellular histones in plasma 24 h and 5 d after blunt chest trauma or sham, * differences to sham procedure were significant, p<0.05; n = 4 for each bar. B. Representative H.E staining of left ventricles 24 h or 5 d after blunt chest trauma or sham procedure as indicated.

With the objective to assess cardiac injury after blunt chest trauma in rats, H.E. staining of left ventricle tissue was performed, revealing no extravasation of erythrocytes in sham-treated animals (0/4). In 2/4 and 3/4 rats at 24 h and 5 d after blunt chest trauma, respectively, extravasation of erythrocytes was present in histological sections (frame B).

### Local inflammation in the left ventricular tissue after trauma (Fig 2)

To assess inflammation, left ventricle tissue homogenates were obtained either 24 h or 5 d after blunt chest trauma or sham procedure. MPO activity, indicating the recruitment and activation of neutrophils, was significantly increased in cardiac tissue 24 h and 5 d after blunt chest trauma (frame A). The expression of the proinflammatory cytokine IL-1β in left ventricles, as assessed by qRT-PCR, was significantly increased 24 h after blunt chest trauma compared to the sham procedure (frame B). C5aR1 protein assessed by western blot was diminished in left ventricles 24 h after blunt chest trauma compared to the sham procedure (frame C). C5aR mRNA expression was not different in sham treated animals compared to blunt chest trauma procedure (frame D).

**Fig 2:**
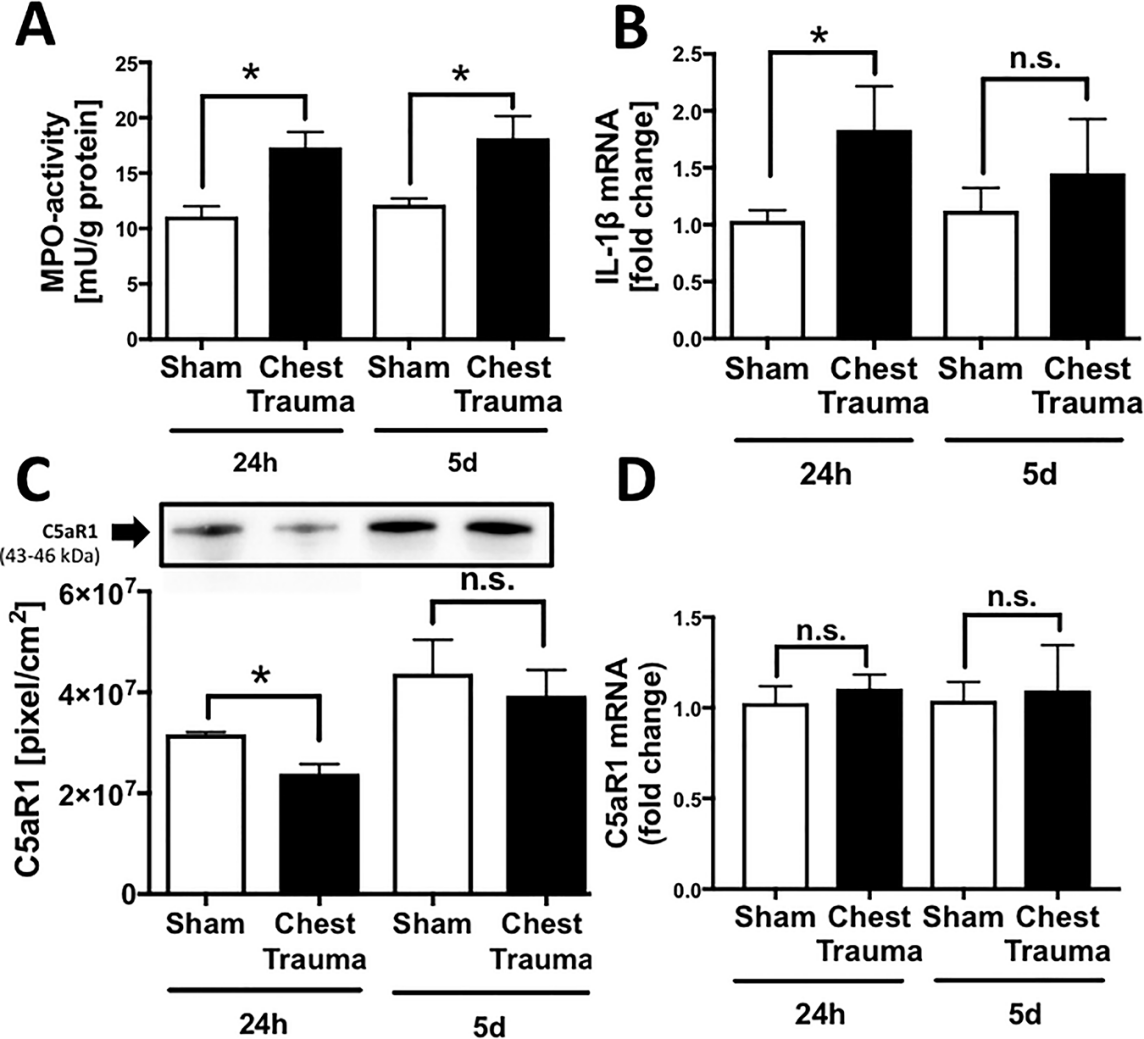
Local inflammation in cardiac injury after blunt chest trauma. A. Increased myeloperoxidase (MOP) activity in left ventricular cardiac tissue 24 h and 5 d after blunt chest trauma compared to sham procedure. B. Elevation of proinflammatory cardiodepressive cytokine IL-1β expression in left ventricular homogenates 24 h after blunt chest trauma compared to sham procedure as assessed by qRT-PCR. C. Representative western blot for C5aR1 of left ventricular tissue homogenates. Densitometry revealed diminished C5aR1 protein expression in left ventricular homogenates 24 h after blunt chest trauma compared to sham procedure. D. C5aR expression in left ventricular homogenates 24 h and 5 d after blunt chest trauma and after sham procedure as assessed by qRT-PCR. p<0.05; *differences were significant to sham procedure, For all frames n = 8 for each bar.

### Translocation of Cx43 after blunt chest trauma (Fig 3)

To determine whether gap-junction proteins in the heart were altered after blunt chest trauma, immunhistochemical staining of Cx43 gap-junction protein in left ventricular tissue sections was performed. In sham-treated animals, Cx43 was located in intercalated discs, whereas 24 h and 5 d after blunt chest trauma, Cx43 was translocated and scattered into the cytosol (frame A). Western-blot analysis provided data showing slightly but not significantly increased Cx43 protein concentrations in left ventricular tissue homogenates 24 h after blunt chest trauma (frame B).

**Fig 3:**
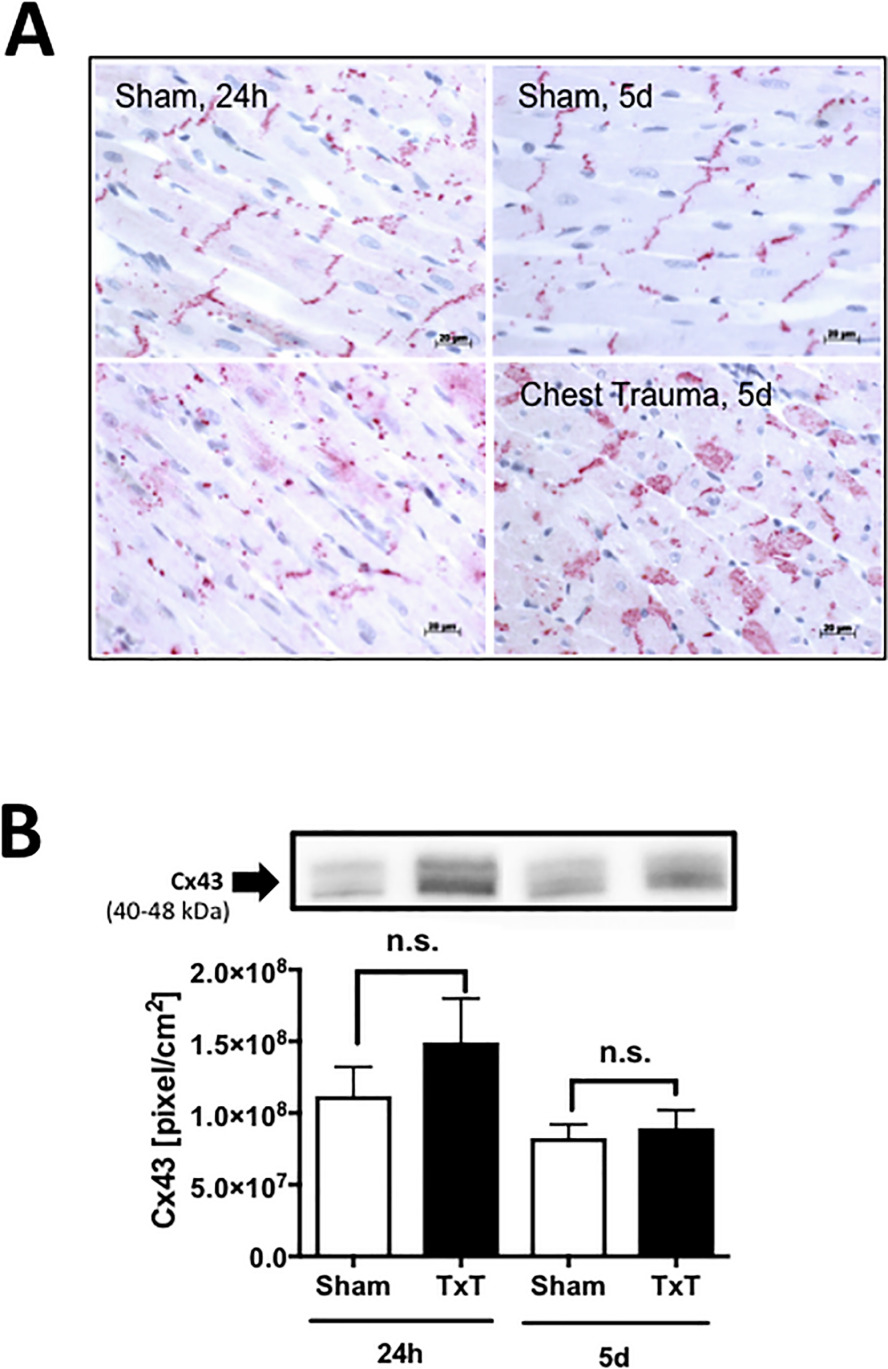
Structural alterations in the heart after blunt chest trauma. Alteration of gap junctional protein connexin 43 (Cx43) after blunt chest trauma in the heart. A. Representative distribution of Cx43 in cardiac tissue after sham procedure or 24 h or 5d after blunt chest trauma as indicated. B. Representative western blot for Cx43 of left ventricular tissue homogenates. Densitometry revealed no significant increase in protein expression in left ventricular homogenates 24 h and 5 d after blunt chest trauma compared to sham procedure. For all frames n = 4 for each bar.

### Apoptosis in cardiac tissue after blunt chest trauma (Fig 4)

Increased apoptosis was observed after blunt chest trauma in the heart 5 d after trauma compared to sham procedure as assessed by immunohistochemical staining of cleaved caspase 3 (frame A). Densitometry provided data showing significantly increased cleaved caspase 3 staining 5 d after blunt chest trauma compared to sham procedure. Trauma induced cardiomyocyte apoptosis was further determined by TUNEL staining in left ventricular tissue. 24 h after trauma apoptosis was slightly increased after trauma compared to sham procedure whereas 5 d after blunt chest trauma apoptosis was significantly increased compared to sham procedure (frame C).

**Fig 4:**
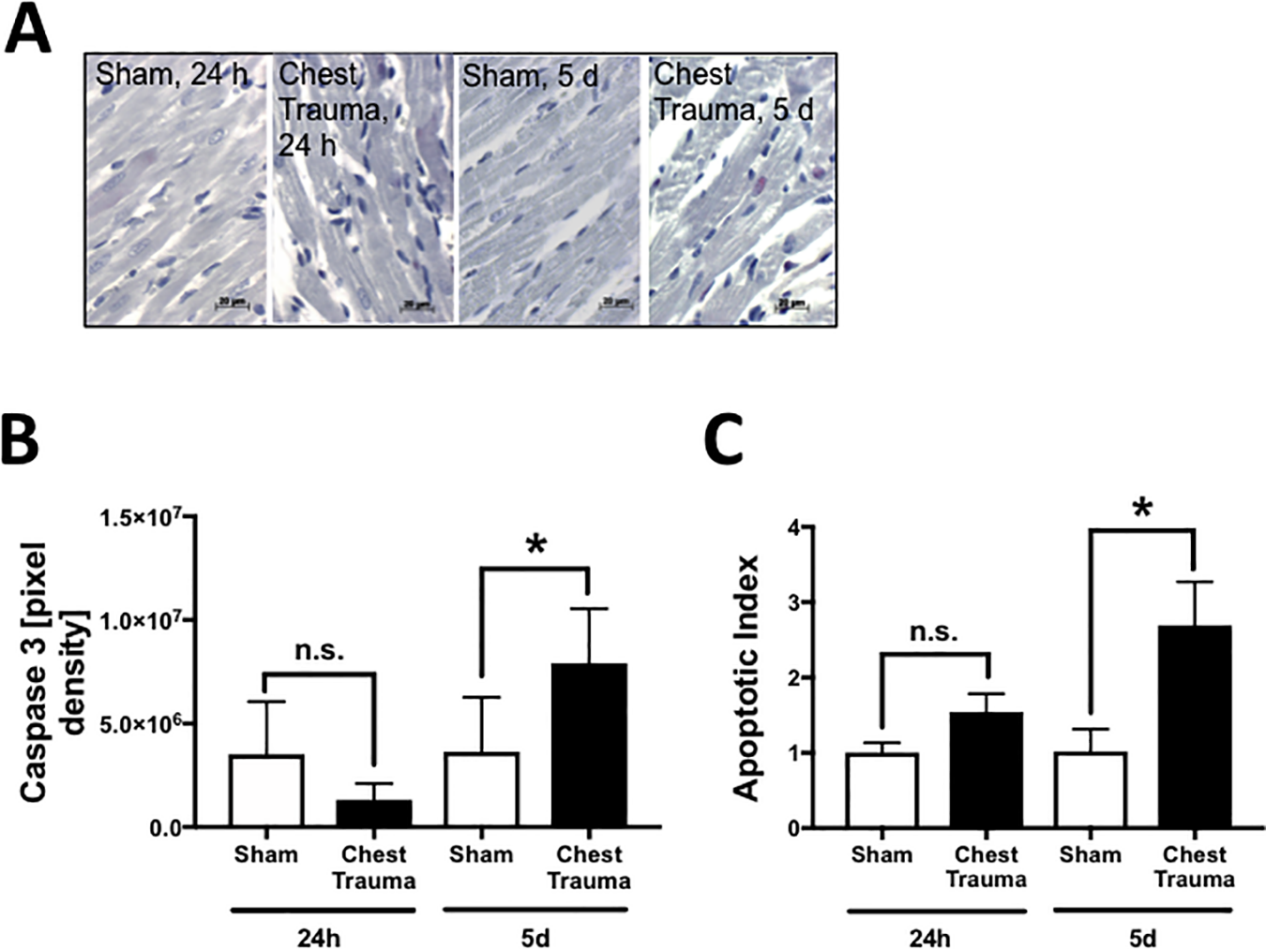
Apoptosis in the heart after blunt chest trauma. A. Representative caspase 3 staining in cardiac tissue after sham procedure or 24 h and 5d after blunt chest trauma as indicated. B. Changes in pixel density of caspase 3 staining in left ventricular tissue 24h or 5d after blunt chest trauma compared to sham procedure as assessed by immunohistochemistry. C. Trauma induced cardiomyocyte apoptosis was determined by TUNEL (TdT-mediated dUTP-biotin nick end labeling) staining in left ventricular tissue 24h and 5d after blunt chest trauma compared to sham procedure as assessed by fluorescence mircoscopy. The total number of nuclei in cardiomyocytes were determined by DAPI staining and apoptotic nuclei were determined by positive TUNEL staining. The apoptotic index was calculated (number of positively stained/total number of nuclei). For all frames n = 4 for each bar. * p<0.05.

Expression of α-actinin, desmin and SERCA in cardiac tissue after blunt chest trauma (S1 Fig)

Altered expression of z-disc proteins after blunt chest trauma was observed in the heart. Desmin expression was diminished 24 h and 5 d after blunt chest trauma compared to the sham procedure (frame A). α2-Actinin expression was likewise reduced 24 h after blunt chest trauma compared to the sham procedure (frame B). To assess alteration of calcium pumps in the heart, SERCA mRNA was determined by qRT-PCR. SERCA expression 24 h after blunt chest trauma was slightly increased (frame C).

## Discussion

We investigated the association between blast wave-induced blunt chest trauma and structural and molecular alterations in cardiac tissue. In animal studies of myocardial contusion, the increase of systemic markers specific for cardiac-cell damage, including cardiac troponins, depends on the power of the kinetic energy applied to the heart [30].

Extracellular histones released after trauma might be important protagonists of cardiac dysfunction. In the present study, we confirmed the appearance of histones in plasma 24 h and 5 d after trauma compared to sham-treated animals. This result is in agreement with earlier studies demonstrating the appearance of circulating histones after trauma in humans and mice [16, 31]. Furthermore, a significant correlation between circulating histone levels and Sequential Organ Failure Assessment (SOFA) [16] was observed in patients with traumatic lung injury. Extracellular histones were found to be major mediators of cardiac injury in patients with sepsis [32] and in experimental murine sepsis [29]. The presence of histones in plasma was associated with their accumulation in the heart [33] in vivo, and on the plasma membrane and in the cytosol of cardiomyocytes in vitro [29]. In earlier studies, we observed a robust increase of intracellular calcium and reactive oxygen species in cardiomyocytes after exposure to extracellular histones [29], which has been linked to defective cardiomyocyte function [34, 35], [36]. Correspondingly, an enhanced extracellular-histone concentration after blunt chest trauma in rats might be linked to cardiac dysfunction.

Histological analysis of the left ventricle tissue after blunt chest trauma revealed evidence of acute tissue damage that occurs early after trauma, probably as a result of the direct mechanical force applied during blunt chest trauma. However, further studies with earlier time points or serial measurements of systemic markers indicating cardiac-cell damage, including heart-type fatty acid-binding protein (H-FABP) are needed to learn more about the dynamics of H-FABP after blunt chest trauma. Apoptosis staining revealed increased apoptosis in cardiac tissue 5 days after trauma.

In the current study, IL-1β expression in left ventricular homogenates was increased 24 h after blunt chest trauma. Recent studies demonstrated that IL-1β release by cardiomyocytes was complement dependent [37, 38]. Therefore, complement activation with the generation of C5a might support cardiac dysfunction after blunt chest trauma by inducing cardio-depressive cytokines. Of note, in the present study, C5aR1 protein was reduced in left ventricular tissue homogenates of rats 24 h after trauma. This is in contrast to earlier studies in rats, which showed increased C5aR1 expression in the heart 24 h after burn injury [39] and on cardiomyocytes obtained from models of experimental sepsis [24]. One possible explanation for the observed post-trauma C5aR1 downregulation could be a trauma-induced, C5a-triggered internalization of the C5aR1 after trauma [40], which is underlined by the lack of downregulation of the C5aR1 on mRNA level. In sepsis, excessive complement activation and C5a generation in animal models and in humans with sepsis was associated with reduced C5aR1 expression [41, 42]. In isolated perfused hearts, C5a-C5aR interaction induced dysfunction of rat cardiomyocytes, resulting in compromised cardiac function [24]. Cleavage of the C5aR1 by neutrophil serine protease would be a further possible explanation for reduced C5aR protein concentration in cardiac tissue after trauma [43]. Accordingly, MPO measurement in left ventricular homogenates indicated increased neutrophil infiltration in cardiac tissue 24 h and 5 d after blunt chest trauma.

In the present study, mechanical damage and local inflammatory response after blunt chest trauma were associated with altered cardiomyocyte cell-to-cell integrity. Cx43 was found to be translocated from the intercalated disc region to the cytosol, which is in agreement with earlier studies, where alteration of Cx43 was found to be associated with both ischemic [44] and non-ischemic [45, 46] cardiac injury. Gap-junction endocytosis of Cx43 was associated with disruption of functional contact between cardiomyocytes and, as consequence, resulted in disruption of the coordinated spread of electrical activation, which was associated with the loss of mechanical and electrical coupling of cardiomyocytes [47, 48], arrhythmia and cardiac dysfunction [49, 50]. The translocation of Cx43 from the intercalated disc region to the cytosol resulting in loss of passage of small molecules and electrical current flow between cardiomyocytes might be an important mechanism of posttraumatic cardiac dysfunction. Further studies are needed to directly link gap junction pathology to cardiac dysfunction after trauma.

Furthermore, structural cardiac proteins located in the Z-lines, including desmin and α-actinin, were down regulated after trauma. Z-disc proteins have been shown to act as responders to stretch and mechanical tension created by hemodynamic demands. Therefore, alterations may contribute to ventricular dysfunction after trauma [51].

Taken together, our results suggest that blast wave-induced blunt chest trauma in rats is associated with cardiac-cell damage, local inflammation, disturbed gap-junction architecture and the presence of extracellular histones in the circulation. Further studies are needed to identify a direct relationship between the presence of extracellular histones occurring after blunt chest trauma and the appearance of defects in cardiac function and structure. It is possible that neutralizing these extracellular histones after trauma might represent an effective strategy for the treatment of patients with cardiac depression after trauma. The blunt chest trauma model, further characterized in the present work, appears to represent a useful small-animal model to prove such therapeutic strategies in vivo.

## Supporting information

S1 Fig Expression of structural proteins and calcium pump in the heart after trauma. Altered expression of z-disc proteins and calcium pump SERCA after blunt chest trauma in the heart. A. Decrease in desmin expression 24 h and 5 d after blunt chest trauma compared to sham procedure. B. Decrease in α2-actinin expression 24 h after blunt chest trauma compared to sham procedure. C. Increased expression of sarcoplasmatic/endoplasmatic reticulum calcium ATPase (SERCA) 24 h after blunt chest trauma compared to sham procedure. For all frames n = 8 for each bar.
(TIFF)

## Abbreviations

ACTN2: actinin alpha 2
ATP2A2: ATPase, Ca^2+^ transporting, cardiac muscle, slow twitch 2
BCA: bicinchoninic acid
C5a: complement component 5a
C3a: complement component 3a
C5aR: complement component 5a receptor
Cx43: connexin 43
DES: desmin
ELISA: enzyme-linked immunosorbent assay
H.E: hematoxylin and eosin
H-FABP: heart-type fatty acid-binding protein
HPRT 1: hypoxanthine phosphoribosyltransferase 1
HRP: horseradish peroxidase
IL-1β: interleukin-1β
IL-6: interleukin-6
MPO: myeloperoxidase
qRT-PCR: real-time quantitative polymerase chain reaction
RIPA: radio-immuno-precipitation assay
SEM: standard error of the mean
SERCA: sarcoplasmatic/endoplasmatic reticlulum calcium-ATPase
SOFA: Sequential Organ Failure Assessment
TBS: tris-buffered saline
TUNEL: TdT-mediated dUTP-biotin nick end labeling
