# Supplementary figures and images for: Experimental blunt chest trauma-induced myocardial inflammation and alteration of gap-junction protein connexin 43
Source: PLoS One. 2017 Nov 9;12(11):e0187270. doi: 10.1371/journal.pone.0187270 (PMC5679619; doi:10.1371/journal.pone.0187270)

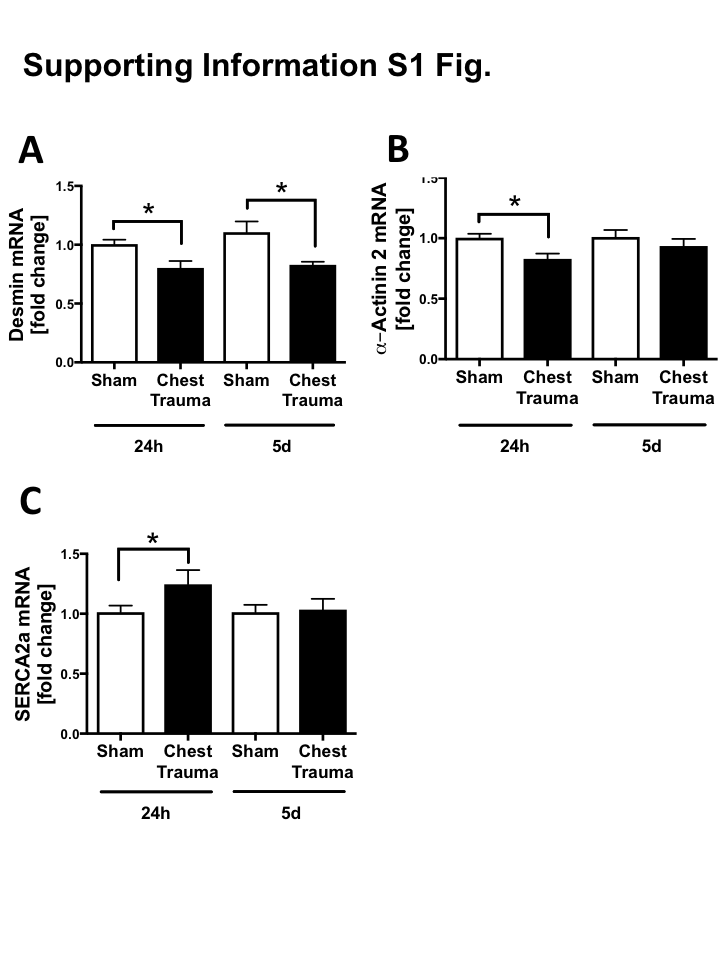

Supplement: S1 Fig — Altered expression of z-disc proteins and calcium pump SERCA after blunt chest trauma in the heart. A. Decrease in desmin expression 24 h and 5 d after blunt chest trauma compared to sham procedure. B. Decrease in α2-actinin expression 24 h after blunt chest trauma compared to sham procedure. C. Increased expression of sarcoplasmatic/endoplasmatic reticulum calcium ATPase (SERCA) 24 h after blunt chest trauma compared to sham procedure. For all frames n = 8 for each bar. (TIFF) [file pone.0187270.s001.tiff]
